# Pulmonary hypertension and right ventricular function in Nigerian children with sickle cell anaemia

**DOI:** 10.1093/trstmh/trz038

**Published:** 2019-05-14

**Authors:** Igoche D Peter, Mustafa O Asani, Shehu U Abdullahi, Ibrahim Aliyu, Stephen K Obaro, Fidelia Bode-Thomas

**Affiliations:** 1 Department of Paediatrics, Aminu Kano Teaching Hospital, Kano, Nigeria; 2 Department of Paediatrics, Bayero University Kano/Aminu Kano Teaching Hospital, Kano, Nigeria; 3 Department of Paediatrics, University of Nebraska Medical Centre, Omaha, NE, USA; 4 International Foundation Against Infectious Diseases in Nigeria, Abuja, Nigeria; 5 University of Jos/Jos University Teaching Hospital, Jos, Nigeria

**Keywords:** children, Nigeria, pulmonary hypertension, sickle cell anaemia, tricuspid annular plane systolic excursion, tricuspid regurgitant velocity

## Abstract

**Background:**

Pulmonary hypertension (PH), a complication of sickle cell anaemia (SCA), results in considerable morbidity. This study aims to determine the prevalence and associations of echocardiography-suggested PH in children with SCA.

**Methods:**

We performed a cross-sectional comparative study involving 100 systematically sampled SCA subjects 3–14 y of age in their steady state with matched haemoglobin AA phenotype controls. Clinical, laboratory and echocardiography data (including tricuspid regurgitation velocity [TRV], mean pulmonary arterial pressure [mPAP] and tricuspid annular plane systolic excursion [TAPSE]) were obtained from all patients. Statistical analyses were performed using SPSS version 22 (IBM, Armonk, NY, USA). A p-value <0.05 was considered statistically significant.

**Results:**

Of the 100 SCA subjects studied, 22 (22%) had echocardiographic findings suggestive of PH compared with none in the controls. The median TAPSE was significantly lower in the PH group (2.55 cm [interquartile range {IQR} 2.2–2.8]) compared with the no PH group (2.77 cm [IQR 2.4–3.2]) (p=0.03). No significant correlation existed between mPAP and age, nor any laboratory parameters studied. The odds ratio (OR) suggested PH significantly increased with an increase in the frequency of hospitalizations for vaso-occlusive crises within a 12-month period (OR 15.15 [95% CI 1.57 to 146.35], p=0.02) and a lifetime history of blood transfusion (OR 5.44 [95% CI 1.09 to 27.24], p=0.04).

**Conclusions:**

Echocardiography-suggested PH is common in children with SCA and is associated with poorer right ventricular function, frequent vaso-occlusive crises and blood transfusions.

## Introduction

Pulmonary hypertension (PH), defined as right heart catheterization-derived mean pulmonary arterial pressure (mPAP) ≥25 mmHg, is an ill-omened long-term complication of sickle cell anaemia (SCA) and is associated with accelerated mortality.^1–3^ It occurs in SCA patients via multifactorial mechanisms such as limited nitric oxide bioavailability and haemolysis, vasculopathic changes, parenchymal injury and thromboembolic disease.^2^ These lead to repeated episodes of hypoxic events characterized by ischaemia–reperfusion injury with progressive tissue damage, alteration of pulmonary vascular tone and vascular proliferation in the arterial smooth muscle wall leading to an elevation of mPAP.

PH has been reported in 40% of the adult SCA population^2^ and available reports on PH in children with SCA have shown a wide prevalence range (10.6–38%).^4–15^ There is, however, a dearth of information on PH in Nigerian children with SCA despite the fact that Nigeria has the largest sickle cell gene pool in the world,^16,17^ and no such study has been conducted in Kano, the state with the largest burden of SCA in the country.^16,18^ The present study therefore aimed to determine the prevalence of echocardiography-suggested PH in children with SCA in Kano in their steady state and to establish its relationship with right ventricular function and clinical and laboratory parameters.

## Methods

### Study location

This study was conducted at the Aminu Kano Teaching Hospital (AKTH), Kano, Nigeria between January and August 2016. Children with SCA were recruited from the paediatric sickle cell clinic and controls were recruited from the paediatric outpatient department of the AKTH.

### Study design

This was a cross-sectional comparative study.

### Study population

Children 3–14 y of age with high-performance liquid chromatography (HPLC)-confirmed haemoglobin SS (HbSS) phenotype and who were in steady state qualified for enrolment into the study.^11^ Steady state was defined as a crisis-free period of at least 3 weeks, with at least 3 months since the last blood transfusion. A comparison group included children with HPLC-confirmed Hb AA phenotype presenting to the outpatient department of the AKTH for medical check-up or minor dermatologic complaints, as well as apparently healthy children accompanying their siblings to the hospital. Children on hydroxyurea therapy, chronic transfusion therapy or those with any chronic or recent (within the preceding 3 weeks) respiratory illness, human immunodeficiency virus (HIV) infection, renal disease or any congenital/acquired heart disease were excluded from the study.

### Sample size determination and sampling method

The minimum number of subjects and controls required for the study was calculated based on a prevalence of 25% obtained in the study by Aliyu et al.^13^ The standard formula for sample size determination in comparative studies was used.^19^ The calculated sample size was approximately 82 for each group, but was increased by 20% to allow for subjects whose tricuspid regurgitant jet velocities were too weak or immeasurable. A total of 100 subjects and 100 controls were therefore systematically sampled (one out of every four eligible patients) for participation in the study per clinic day.

### Data collection

An interviewer-administered pro forma designed specifically for the study was used to collect information (biodata, clinical history and physical examination including anthropometry, laboratory and echocardiography findings) from both subjects and controls. Echocardiography was done by one of the authors (IDP) using an SSI-8000 cardiac ultrasound system (SonoScape, Shenzhen, China). For quality control, 10% of the echocardiograms were independently validated by the second author (MOA).

Cardiac measurements were performed according to the guidelines of the American Society of Echocardiography.^20^ Right atrial pressure (RAP) was estimated from the percentage inferior vena cava (IVC) collapsibility index after the IVC diameter was determined using M-mode at normal expiration then at full inspiration.^21^ Tricuspid regurgitation was assessed twice each in the apical four-chamber, parasternal short axis and the modified parasternal long axis (showing the right ventricular inflow tract) views. The peak tricuspid regurgitant velocity (TRV) obtained was recorded along with the corresponding peak pressure gradient derived using the modified Bernoulli equation.^22^ Pulmonary artery systolic pressure (PASP) was estimated by adding the generated peak tricuspid regurgitant pressure gradient to the estimated RAP. By applying the formula of Chemla et al.^23^ ([0.61×PASP]+2 mmHg), the mean pulmonary artery pressure (mPAP) was derived for each subject. The prevalence of echocardiography-suggested PH was evaluated using two criteria: TRV ≥2.5 m/sec^9–14^ and mPAP ≥25 mmHg.^1^ The choice of the second criterion (mPAP ≥25 mmHg) was based on the recommended definition of PH (which is regardless of Hb phenotype) at the 4th World Symposium on PH in 2008 (Dana Point, CA, USA).^1^ Using echocardiography, multiple measurements (RAP, TRV and PASP) were obtained and mPAP was derived from them. Other supportive echocardiographic evidence of PH were sought in accordance with the 2015 diagnostic guidelines of the European Society of Cardiology/European Respiratory Society.^24^ These include abnormal positioning of the interventricular septum, IVC collapsibility index <50% and dilatation of the main pulmonary artery.^24^ The tricuspid annular plane systolic excursion (TAPSE) was acquired by placing the M-mode cursor through the lateral tricuspid annulus and measuring the amount of longitudinal motion of the annulus at peak systole.^25^

#### Laboratory investigations

Full blood count was done using the Swelab Alpha Automated haematology system (Boule Medical, Stockholm, Sweden), reticulocyte counts were manually obtained and HIV screening was done using the Determine HIV 1/2 Test Kit (Alere Medical, Tokyo, Japan). Total serum lactate dehydrogenase (LDH) activity in serum was measured using the in vitro quantitative kinetic method, while the Hb phenotypes and foetal haemoglobin (HbF) levels of all study participants were determined using the Variant II Hemoglobin Testing System (Bio-Rad, Hercules, CA, USA).

### Statistical analysis

Data was analysed using the Statistical Package for Social Sciences (SPSS) software, version 22 (IBM, Armonk, NY, USA). Continuous variables were assessed for normality using the Kolmogorov–Smirnov test and expressed as mean±standard deviation (SD) for continuous/normally distributed data or median (interquartile range [IQR]) for non-normally distributed data. The Student t test or Mann–Whitney U test, as necessary, was used to compare parameters between the SCA and control groups and between the PH and non-PH groups of children with SCA. The correlation of laboratory parameters and patient age with mPAP was examined using Spearman’s rank order correlation (as these data were all non-normally distributed). The χ^2^ or Fisher’s exact test (where necessary) were used to determine the association between PH and the clinical characteristics of children with SCA. Logistic regression analysis was used to determine predictors of PH. A p-value <0.05 was considered significant for all tests.

## Results

### Demographic characteristics

A total of 100 children with SCA and an equal number of age- and sex-matched controls were enrolled in this study. There were 54 male and 46 female subjects and controls, for a male:female ratio of 1.2:1. The respondents’ median age was 7 y (IQR 7–11). Both subjects and controls were similar with respect to mean height, weight and pulse rate, but transcutaneous oxygen saturation was significantly lower in the SCA group (Table 1).

**Table 1.** Comparison of some basic anthropometric and clinical parameters of 100 children with SCA and 100 age- and sex-matched controls

Parameter	SCA subjects	Hb AA controls	Test statistic	p-Value
Height (cm), mean±SD	118.64±16.0	121.41±19.7	−1.08^a^	0.28
Pulse (beats/min), mean±SD	101.53±15.6	101.22±18.5	0.13^a^	0.90
Oxygen saturation (%), median (IQR)	97.00 (93.0–98.0)	99.00 (98.0–99.0)	−6.62^b^	0.0001*
Weight (kg), median (IQR)	20.00 (18.0–27.0)	21.00 (17.0–28.3)	−0.46^b^	0.65

BSA: body surface area.

^a^Student t test.

^b^Mann–Whitney U test.

*Statistically significant.

### Clinical and laboratory parameters

Acute painful crisis was the most common cause of hospitalization in the 12 months prior to enrolment into this study, and this occurred more than once in 10% of the study population. Other reasons for hospitalization over the same time period included severe anaemia requiring blood transfusion, stroke and priapism (Table 2).

**Table 2.** Clinical history of 100 children with SCA

Clinical events	Frequency, n (%)
Lifetime	
Hospitalizations	72 (72.0)
Acute painful crisis	53 (53.0)
Obtained blood transfusion	45 (45.0)
Acute chest syndrome/pneumonia	6 (6.0)
Stroke	3 (3.0)
Priapism (in 54 males)	3 (5.6)
Preceding 12 months	
Acute painful crises	
1	16 (16.0)
≥2	10 (10.0)
Blood transfusion	
1	10 (10.0)
≥2	5 (5.0)
Stroke	
1	1 (1.0)
≥2	0 (0.0)
Priapism (in 54 males)	
1	2 (3.7)
≥2	0 (0.0)

Children with SCA were significantly different from the controls with respect to the presence of pallor and jaundice (p=0.000). The most common physical abnormality in the SCA subjects was pallor (57%), followed by jaundice (37%). In only one SCA patient was digital clubbing observed, and this was grade 2.

SCA subjects had a significantly lower mean haematocrit but higher mean total white blood cell (WBC), platelet and reticulocyte counts, as well as higher mean HbF and LDH enzyme levels when compared with controls (Table 3).

**Table 3.** Laboratory parameters of 100 children with SCA and 100 Hb AA controls

Laboratory parameter	SCA subjects, median (IQR)	Hb AA controls, median (IQR)	Test statistic^a^	p-Value
LDH level (IU/l)	806.00 (643.5–886.3)	493.00 (345.0–677.5)	−6.68	<0.0001*
Platelet count (×10^9^/l)	371.00 (257.0–467.5)	286.00 (240.8–335.5)	−4.03	<0.0001*
Haematocrit (%)	22.45 (20.2–24.4)	33.20 (31.2–36.6)	−11.78	<0.0001*
Total WBC count (×10^9^/l)	13.35 (10.5–16.1)	6.20 (5.2–7.3)	−11.17	<0.0001*
Reticulocyte count (×10^9^/l)	11.00 (8.0–15.6)	1.80 (1.1–2.1)	−11.95	<0.0001*
HbF level (%)	9.20 (5.0–14.4)	0.60 (0.4–1.0)	−12.01	<0.0001*
Lymphocyte count (×10^9^/l)	6.25 (4.4–7.8)	3.40 (2.7–4.0)	−9.42	<0.0001*
Granulocyte count (×10^9^/l)	5.65 (4.3–7.7)	2.50 (1.8–3.2)	−10.69	<0.0001*

IU/l: international units per litre.

^a^Mann–Whitney U test.

*Statistically significant.

### Two-dimensional and M-mode echocardiography

All the cardiac chambers were significantly larger in SCA patients, as were the diameters of their atrioventricular valve annuli and main pulmonary arteries compared with Hb AA controls (p<0.0001). The thickness of the right ventricular anterior wall was similar in both the cases and controls (Table 4).

**Table 4.** Echocardiographic measurement of some right cardiac parameters in 100 SCA cases and 100 age- and sex-matched controls

Dimension	SCA subjects	Hb AA controls	Test statistic	p-Value
RA area (mm^2^), mean±SD	767.16±204.4	626.35±196.5	4.95^a^	<0.0001*
TV annulus diameter (mm), mean±SD	25.60±3.9	21.62±3.8	7.2^a^	<0.0001*
MPA diameter (mm), median (IQR)	16.54 (15.1–18.4)	15.06 (13.8–16.4)	−4.59^b^	<0.0001*
RVIDd (cm), mean±SD	2.54±0.5	2.27±0.4	2.44^a^	0.02*
RVAWTd (cm), median (IQR)	0.33 (0.3–0.4)	0.33 (0.3–0.4)	−1.33^b^	0.18

MPA: main pulmonary artery; RA: right atrium; RVAWTd: right ventricular anterior wall thickness in diastole; RVIDd: right ventricular internal dimension in diastole; TV: tricuspid valve.

^a^Student t test.

^b^Mann–Whitney U test.

*Statistically significant.

### PAP-related parameters

Tricuspid valve regurgitation was present in 90% of cases and 89% of controls. The TRV of the SCA group ranged from 1.1 to 3.8 m/sec while that of the control group ranged from 1.02 to 2.44 m/sec; hence 22 SCA subjects had a TRV ≥2.5 m/sec, while all controls had values <2.5 m/sec. The SCA group had a median TRV of 2.21 m/s (IQR 2.0–2.6), and this was significantly faster than that of the controls, which was 2.01 m/s (IQR 1.8–2.18) (p<0.0001). The mPAP was significantly elevated in SCA subjects compared with controls (Table 5).

**Table 5.** pulmonary artery pressure parameters of 90 SCA cases and 89 age- and sex-matched controls with tricuspid valve regurgitation

Parameter	SCA subjects, median (IQR) [range]	Hb AA controls, median (IQR) [range]	Test statistic^a^	p-Value
PASP (mmHg)	30.03 (26.2–37.4) [14.5–68.8]	26.00 (23.4–29.4) [9.2–34.0]	−4.23	<0.0001*
mPAP (mmHg)	20.32 (17.9–25.0) [10.8–44.0]	17.86 (16.3–19.9) [7.6–22.7]	−4.21	<0.0001*
Tricuspid PPG (mmHg)	19.68 (16.0–26.1) [4.5–58.8]	16.10 (13.4–19.0) [4.2–24.0]	−4.18	<0.0001*
TR jet length (mm)	16.77 (11.9–22.7) [1.5–87.5]	11.13 (5.8–16.9) [0.6–31.9]	−4.19	<0.0001*
RAP (mmHg)	10.00 (10.0–10.0) [5.0–15.0]	10.00 (10.0–10.0) [5.0–15.0]	−2.08	0.04*
TRV (m/s)	2.21 (2.0–2.6) [1.1–3.8]	2.01 (1.83–2.18) [1.02–2.44]	−4.19	<0.0001*
TR jet VC width (mm)	2.49 (2.0–3.2) [1.3–5.5]	2.49 (2.1–3.7) [0.8–5.9]	−0.67	0.5

PPG: peak pressure gradient; TR: tricuspid regurgitation; VC: vena contracta.

^a^Mann–Whitney U test.

*Statistically significant.

Using both criteria for the definition of echocardiography-suggested PH (TRV ≥2.5 m/s and mPAP ≥25 mmHg), the prevalence of echocardiography-suggested PH in children with SCA was 22%. None of the controls had PH, as their TRVs ranged from 1.02 to 2.44 m/s (which is <2.5 m/s) and their mPAP ranged from 7.6 to 22.7 mmHg (also <25 mmHg). Fifteen of the 22 children with SCA with PH were males and 7 were females. PH was most common among children 3–6 y of age (45.5%) compared with 22.7% in the 7–10 y age group and 31.8% in those 11–14 y.

Additional echocardiographic evidence of PH^24^ was present in 18 of the 22 (81.8%) SCA with PH patients (Table 6). A flattening or leftward bulging interventricular septum was the most common of these and was present in 33.3% of the subjects.

**Table 6.** Frequency of additional echocardiographic features of PH in 18 SCA subjects with PH

Parameter	Frequency, n (%)
Flat/left bulging IVS only	6 (33.3)
Dilated MPA+flat/left bulging IVS	5 (27.8)
IVC collapsibility index <50% only	3 (16.6)
IVC collapsibility index <50%+flat/left bulging IVS	2 (11.1)
Dilated MPA+IVC collapsibility index <50%	1 (5.6)
Dilated MPA alone	1 (5.6)
Total present	18 (100)

IVS: interventricular septum; MPA: main pulmonary artery.

SCA subjects with echocardiography-suggested PH generally had larger intracardiac dimensions than those without, but the differences were not statistically significant (Table 7). TAPSE was significantly lower in the SCA subjects with PH compared with those without PH, although both categories had median TAPSE values within the normal range for their ages when compared with published normative values.^25^

**Table 7.** Echocardiographic measurement of some right cardiac parameters in SCA subjects with and without PH

Parameter	PH group (n=22)	No PH group (n=78)	Test statistic	p-Value
RA area (mm^2^), mean±SD	821.91±222.9	751.73±197.6	1.43^a^	0.16
TV annulus diameter (mm), mean±SD	25.57±3.7	25.61±4.06	−0.05^a^	0.97
MPA diameter (mm), median (IQR)	17.11	16.48	−1.59^b^	0.11
	(15.9–20.5)	(14.6–18.14)		
RVIDd (cm), mean±SD	2.58±0.7	2.53±0.4	0.37^a^	0.71
TAPSE (cm), median (IQR)	2.55	2.77	−2.22^b^	0.03*
	(2.2–2.8)	(2.4–3.2)		
RVAWTd (cm), median (IQR)	0.33	0.33	−1.57^b^	0.12
	(0.28–0.33)	(0.28–0.44)		

MPA: main pulmonary artery; RVAWTd: right ventricular anterior wall thickness in diastole; RVIDd: right ventricular internal dimension in diastole; TV: tricuspid valve.

^a^Student t test.

^b^Mann–Whitney U test.

*Statistically significant.

### Comparison of some clinical and laboratory parameters between SCA subjects with and without echocardiography-suggested PH

SCA subjects with echocardiographic features suggestive of PH were older and had lower haematocrit levels than their counterparts without PH. They also had lower HbF and LDH levels, but higher reticulocyte, platelet and WBC counts compared to their counterparts without PH (Table 8). None of these observed differences was statistically significant. No significant correlation existed between mPAP and age, nor any laboratory parameters studied.

**Table 8.** Laboratory parameters of SCA cases with and without PH

Parameter	PH group (n=22), median (IQR)	No PH group (n=78), median (IQR)	Test statistic^a^	p-Value
LDH level (IU/l)	759.50 (533.5–877.0)	810.00 (672.0–887.3)	−0.69	0.49
Platelet count (×10^9^/l)	377.00 (268.8–476.8)	367.50 (254.0–466.0)	−0.47	0.64
Haematocrit (%)	21.45 (19.8–23.1)	22.7 (20.3–25.2)	−1.52	0.13
Total WBC count (×10^9^/l)	14.15 (11.6–16.9)	13.0 (10.1–15.5)	−1.33	0.18
Reticulocyte count (×10^9^/l)	11.1 (8.6–14.1)	11.00 (7.6–16.0)	−0.08	0.93
Age (years)	7.50 (4.8–11.3)	7.00 (6.0–11.0)	−0.27	0.79
HbF level (%)	7.2 (3.8–13.7)	9.75 (5.4–14.5)	−0.46	0.65

IU/l: international units per litre.

^a^Mann–Whitney U test.

### Association of echocardiography-suggested PH with the number of clinical events in SCA subjects in the preceding 12 months

The number of blood transfusions and episodes of acute painful crises per subject in a 12-month period was more frequent in SCA subjects with echocardiography-suggested PH compared with those without PH (p=0.007 and p<0.0001, respectively). Within a 12-month period, children with SCA who were hospitalized once for an acute painful crisis had 14.44 times greater odds of having PH, and this increased to 15.15 times in those with two or more hospitalizations for crises. Those who had ever received a blood transfusion had 5.44 times greater odds of having PH compared with those who had never been transfused (p=0.04) (Table 9).

**Table 9.** Adjusted ORs with 95% CIs for logistic regression models used to determine predictors of PH

Clinical events	Adjusted OR (95% CI)	p-Value
Frequency of Acute painful episodes in the last 12 months		
None^a^	1.00	
1	14.44 (2.38 to 87.53)	0.004*
≥2	15.15 (1.57 to 146.35)	0.02*
Frequency of blood transfusion in last 12 months		
None^a^	1.00	
1	2.40 (0.11 to 52.02)	0.56
≥2	2.13 (0.065 to 69.88)	0.67
Lifetime history of blood transfusion		
None^a^	1.00	
Ever received	5.44 (1.09 to 27.24)	0.04*

^a^Reference group.

*Statistically significant.

## Discussion

PH as suggested by echocardiography was present in more than one in every five children with SCA in the present cohort but was absent in the controls. The prevalence of PH in the present study (22%) is strikingly similar to the 22.9% reported in children with SCA by Sokunbi et al.^12^ in Lagos (southwest Nigeria) and the 25% reported in a cohort of children and adults with SCA by Aliyu et al.^13^ in Zaria (northern Nigeria). This is despite the fact that these earlier studies inferred PH from TRV, while we additionally did so from mPAP, which was derived from a composite of the RAP, TRV and PASP using the formula of Chemla et al.^23^ Interestingly, defining PH from echocardiography on the basis of TRV and mPAP gave the same prevalence of PH in our study cohort. It is known however that mPAP can be dissimilar if patients with similar TRVs have different RAP values. Hence the similarity in prevalence of echocardiography-suggested PH despite different definition criteria may be reflective of similarity in RAP values.

We found SCA subjects without PH had significantly better right ventricular function than those with echocardiography-suggested PH (p=0.03). Zakaria et al.^26^ similarly observed that TAPSE was significantly lower in their cohort of 30 children with PH after matching with controls by age. Since right ventricular function is an important prognostic determinant of cardiopulmonary pathologies in children,^27^ our observation of lower TAPSE in SCA children in the PH group portends a poor prognosis. This is because as pulmonary pressure increases, the right ventricle progressively dilates, culminating in right ventricular systolic dysfunction and terminal failure.

Children with SCA who had echocardiography-suggested PH did not differ from those without PH with respect to age (p=0.79), and this finding concurs with those of most other researchers.^5,6,8,11,13^ In the present study, echocardiographic features of PH occurred as early as 3 y of age in one child and 4 y in four children, similar to the study by Colombatti et al.^11^ in Italy, which found PH in three children 3 y of age, and another study by Minniti et al.^6^ in the USA that reported PH in a 3-year-old child. It is therefore plausible that the pathophysiologic changes culminating in PH that are well reported in adults start early in life, probably before the age of 3 y in children with SCA, hence future studies should be done at earlier ages.

It was also observed that SCA children without PH had higher HbF levels than those with echocardiography-suggested PH and it is therefore likely that HbF offers some protection, although this difference was not statistically significant. A similar observation was made by Sokunbi et al.^12^ in Lagos, Al-Allawi et al.^5^ in Iraq and Colombatti et al.^11^ in Italy. Although HbF is the major genetic modulator of the haematologic and clinical features of sickle cell disease, it appears that high levels of HbF may not completely protect against PH in children with SCA. This is unclear and will require further studies.

We found that the haematocrit levels of SCA subjects with echocardiography-suggested PH were not different from the levels in those who did not have PH (p=0.13), in agreement with Sokunbi et al.^12^ It is conceivable that with the lower haematocrit levels in SCA, cardiac output increases in a bid to catch up with metabolic demands.^9^ However, even though cardiac output may increase, measured intracardiac velocities have not been shown to significantly increase in SCA subjects with echocardiography-suggested PH.

Although, as expected, reticulocyte counts were significantly higher in children with SCA compared with controls (p<0.0001), there was no significant difference in reticulocyte counts between children who had PH and those who did not (p=0.93). This is in tandem with findings by Aliyu et al.^13^ but contradicts the findings by Pashankar et al.,^14^ Minniti et al.^6^ and Colombatti et al.,^11^ who included HbSS, HbSβ thalassaemia and HbSC in their study population rather than strictly HbSS patients as in ours and that of Aliyu et al.^13^ Their mixed spectrum of sickle cell disorders inevitably exerts a confounding influence of varying but undefined degree upon interpretability in terms of explaining pathophysiology.^28^

The correlation of total WBC counts with mPAP, though positive, was weak and not significant in SCA subjects, both in those with and without PH. This agrees with the findings of Dosunmu et al.^29^ and Aliyu et al.^13^ Understandably, as an inclusion criterion for these studies, study participants were free of any clinical event (including fever, painful episodes, acute chest syndrome or hospital admission) for a period of at least 3 weeks prior to enrolment.

Whereas SCA subjects had higher platelet counts compared with the controls, there was no significant difference in the platelet counts of SCA patients with or without PH, agreeing with work by Al-Allawi et al.^5^ and Aliyu et al.^13^ The actual difference between the PH and no PH groups may be primarily in terms of platelet function, as activated platelets play a direct role in the development of microthrombi, which worsen PH in patients with SCA. There are currently no facilities for assessing platelet function in the study centre.

In the present study, total serum LDH did not correlate with mPAP and the LDH levels of SCA children with PH were similar to those with normal pulmonary pressure (p=0.49). This agrees with some reports^6,30^ but differs from that of Lee et al.,^10^ who reported significantly higher LDH levels in SCA children with PH compared with those without PH. Their study population differs from ours in that they had sickle cell patients with HbSβ^0^ thalassemia, HbSβ^+^ thalassemia and hereditary persistence of HbF in addition to the homozygous SS population, and LDH values are known to vary among SCD phenotypes.^28^

The marked increase in the odds of developing echocardiographic features of PH among SCA patients who were hospitalized for acute painful crises in the preceding 12 months or had ever been transfused is consistent with the findings of Al-Allawi et al.^5^ and Minniti et al.^6^ The association with blood transfusion requirement is not a surprise, as this is a hallmark of sickle cell vasculopathy, which occurs in PH.^30^

## Conclusions

The prevalence of echocardiography-suggested PH in children with SCA in their steady state is similar to reports from other parts of Nigeria. Clinical associations of PH in children with SCA include a lifetime history of ever receiving a blood transfusion, a preceding 12-months history of having received two or more blood transfusions and experiencing at least one acute painful episode requiring hospitalization in the preceding 12 months. Right ventricular function was poorer in SCA subjects with echocardiography-suggested PH.

Based on our observations, we recommend that children ≥3 y of age with SCA who have received two or more blood transfusions and/or had two or more acute painful crises requiring hospitalization within a given 12-month period should have an echocardiographic assessment for PH. Furthermore, routine care of children with SCA should be optimized so as to minimize episodes of severe anaemia requiring blood transfusion and the frequency of severe acute painful crises warranting hospitalization, as these were found to be associated PH.
